# Antioxidant Activity and Phenolic Content of *Paederia foetida* and* Syzygium aqueum*

**DOI:** 10.3390/molecules14030970

**Published:** 2009-03-03

**Authors:** Hasnah Osman, Afidah A. Rahim, Norhafizah M. Isa, Nornaemah M. Bakhir

**Affiliations:** Universiti Sains Malaysia, 11800 Gelugor, Pulau Pinang, Malaysia

**Keywords:** *Paederia foetida*, *Syzygium aqueum*, Antioxidant activity, Phenolic content.

## Abstract

The antioxidant activity of fresh and dried plant extracts of *Paederia foetida* and *Syzygium aqueum* were studied using β-carotene bleaching and the 2,2’-azinobis(3-ethyl-benzothiazoline-6-sulfonic acid) (ABTS) radical cation assay. The percentage of antioxidant activity for all extract samples using both assays was between 58 and 80%. The fresh samples of both plants had higher antioxidant activity than the dried samples. The results of the β-carotene bleaching assay were correlated (R^2^ = 0.9849) with those of the ABTS assay.

## Introduction

Plants consumed by humans may contain thousands of different phenolic compounds. The effect of dietary phenolics are of great current interest, due to their antioxidative and possible anticarcinogenic activity [1]. Phenolic compounds also function as free-radical scavengers, reducing agents, and quenchers of singlet-oxygen formation [2]. Antioxidant compounds that scavenge free radicals help protect against degenerative diseases [3]. 

*Paederia foetida* L. (*P. foetida*) is locally known in Malaysia as *akar sekuntut*. This aromatic climbing plant is a leafy vegetable that can be eaten raw or steamed [4]. This popular plant is used as a remedy for diarrhoea and dysentery in Bangladesh [5] and to inhibit intestinal motility [6]. Iridiod glycosides, paederolone, paederone, paederine and paederenine were the phytochemicals identified in this plant [5,7]. Previous studies [7,8] also identified a number of steroids and terpenoids and 77 constituents in the volatile oils of the leaves, stems and flowers of *P. foetida*, some at high levels. 

*Syzygium aqueum* (*S. aqueum*), also known as watery rose apple or water apple, has thirst-relieving properties and is usually consumed raw. In Malaysia the powdered dried leaves are used to treat a cracked tongue and a preparation from the root is used to relieve itching and to reduce swelling [9]. The volatile oils isolated by vacuum distillation from *Syzygium* species contain a high percentage of terpenoids and γ-terpinene [10], with tannins and related compounds are also found in the leaves of *Syzygium* species [11]. Even though there are reports on the antioxidant activity of *P. foetida* and *S. aqueum,* different analytical methods were used [12,13]. Therefore, this study was undertaken to evaluate the antioxidant capability of these plants using two methods: coupled oxidation of β-carotene-linoliec acid and an ABTS assay. The comparison on the percentage of antioxidant activity of fresh and dried samples would also be studied. 

## Results and Discussion

### Extraction

The percentage of the crude extracts in methanol was between 8.8 and 10.1% w/v and they were used in its form after dilution. Methanol was used as the extraction solvent for fresh and dried samples of *P. foetida* and *S. aqueum* due to its polarity and its known ability to extract compounds such as phenolics, flavonoids and other polar materials [14]. 

### Antioxidant activity

### Coupled oxidation of β-carotene and linoleic acid

**Figure 1:**
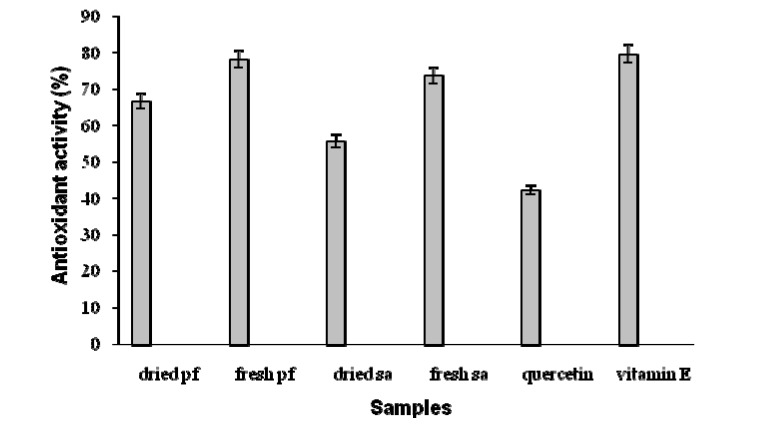
Antioxidant activity of the different extracts and standard samples at 0.02 ppm in the β-carotene-linoleate system. Variations were significant at level p<0.05.

In the β-carotene-linoleic acid coupled oxidation model system, the linoleic acid free radical (LOO**^·^**) formed attacks the highly unsaturated β-carotene molecules and in the absence of an antioxidant rapidly bleaches the typically orange colour of β-carotene which is monitored spectrophotometrically at 450 nm. The extracts reduced the extent of β-carotene bleaching by neutralising the linoleate-free radical and other free radicals formed in the system [15]. The total antioxidant activities of the crude extracts of DL-α-tocopherol, fresh *P. foetida*, fresh *S. aqueum*, dried *P. foetida*, dried *S. aqueum*, and quercetin after 160 h reaction time were 79.69 ± 3.16%, 78.13 ± 2.90%, 73.77 ± 2.95%, 66.67 ± 3.30%, 55.73 ± 2.82% and 42.37 ± 3.25%, respectively (Figure 1). Variations were significant (p<0.05). The fresh samples had higher antioxidant activity than did the dried samples. In this study, the order of antioxidant activity towards β-carotene oxidation was DL-α-tocopherol > fresh *P. foetida* > fresh *S. aqueum* > dried *P. foetida* > dried *S. aqueum* > quercetin. The antioxidant activity of fresh *P. foetida* was the highest and was comparable to DL-α-tocopherol. All of the tested samples more efficiently slowed the bleaching of β-carotene than did quercetin.

pf = *Paederia foetida*  sa = *Syzygium aqueum*

### ABTS free radical-scavenging activity

**Figure 2:**
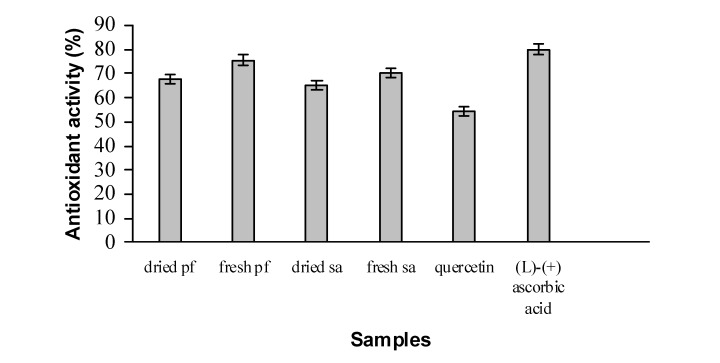
Values of free radical-scavenging activity of sample extracts in ABTS assay. Variations were significant at level p<0.05.

ABTS assay is commonly used to assess radical scavenging or antioxidant activity. The scavenging activity is measured by the absorbance at 414 nm, which decreased as the ABTS radical is scavenged. The free radical scavenging activity of fresh and dried extracts along with reference standards, such as quercetin and (*l*)-(+) ascorbic acid were determined by ABTS assay and the results are shown in Figure 2. All of the extracts had strong antioxidant abilities that exceeded the control, quercetin except (*l*)-(+) ascorbic acid. The difference in the antioxidant activity profiles of the various extracts is consistent with previous reports [16] of different constituents in the extracts. Phenolic compounds scavenge free radicals by forming a stable ABTS-H. The scavenging activity of the extracts also could be due to the presence of steroids and terpenoids which are known to occur in *P. foetida* plant [7,8]. 

**Figure 3:**
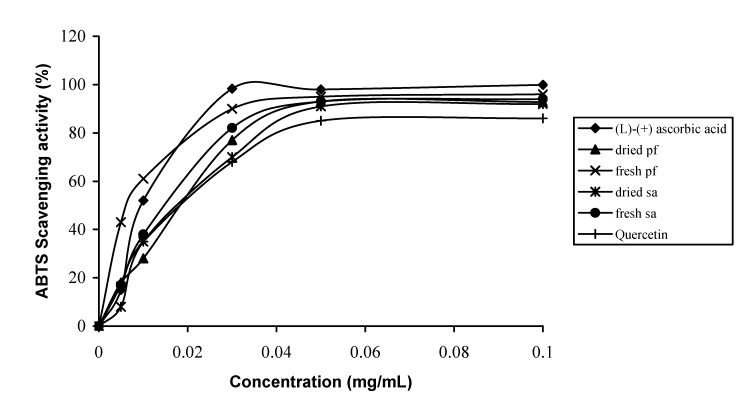
Free radical-scavenging activity of extract samples at different concentration using ABTS ‘assay’. (*l*)-(+) Ascorbic acid and quercetin were used as references.

*P. foetida* has higher levels of phenolic compounds than does *S. aqueum*,which may have contributed to its high antioxidant activity [6,9,17,18]. The fresh *P. foetida* and *S.* *aqueum* extracts had 70-76% antioxidant activity and the dried samples had 65-68% antioxidant activity (Figure 2) which was higher than the activity of the standard commercial antioxidant, quercetin. The decrease in antioxidant activity in the dried samples could be due to degradation of the antioxidants during drying. The storage, processing and preparation conditions are known to alter the content of antioxidants but little information is known about the impact of drying on the antioxidant activity of vegetables [19]. Scavenging activity increased with the extracts concentration (Figure 3). At 0.035 mg mL^-1^, the order of scavenging activity extracts was: fresh *P. foetida* > fresh *S. aqueum* > dried *P. foetida* > dried *S. aqueum*. A plateau was reached at 0.055 mg mL^-1^ with the scavenging activity > 90% for all extracts.

### Total phenolic content of the plant extracts

The total phenolic content for all of the extracts decreased after drying (Table 1). The amount of total phenolics varied widely between 20.80 and 63.00 mg/g sample weight. Previous investigations showed that *P. foetida* and * S. aqueum* are low in total phenolic content compared to other fruit and leaf samples of several plants[12,13]. Total phenolic content in ferulic acid equivalent gave the highest levels of 62.64 ± 1.32 and 60.93 ± 3.4 mg/g sample weight for the fresh leaves and twigs of *P. foetida*, respectively. *P. foetida* leaves and twigs often are consumed raw [4], and the total phenolic content of the twigs was slightly lower than that of the leaves, (Table 1). It is expected that the total antioxidant activity of the twigs should be as good as the leaves sample since antioxidant activity increases proportionally with the phenolic content [1,20]. 

**Table 1:** Total phenolic contents of *P. foetida and S. aqueum.*

Samples	FA^b^
*P. foetida* leaves (dried)	35.52 ± 1.64
*P. foetida* leaves (fresh)	62.64 ± 1.32
*S. aqueum* leaves (dried)	20.77 ± 0.34
*S. aqueum* leaves (fresh)	52.96 ± 1.62
*P. foetida* twig (dried)	20.8 ± 3.25
*P. foetida* twig (fresh)	60.93 ± 3.40

All analyses were mean of triplicate measurements ± standard deviation ^b^Results expressed in mg ferulic acid equivalent/g sample weight

### Correlation between two methods of antioxidant activity

Extracted samples from leaves at a concentration of 0.02 ppm were chosen to test the correlation between the two methods. The percentage of antioxidant activity were between 65 and 80% for both methods (Table 2). Fresh *P. foetida* and *S. aqueum* both had better antioxidant activity than the dried samples. The correlation between the β-carotene oxidation and ABTS methods had R^2^ = 0.9878.

**Table 2:** Comparison of oxidation (%) determination between oxidation β-carotene and ABTS method

Samples	Antioxidant (%)(β-carotene) method	Antioxidant (%)(ABTS) method
*S. aqueum* Leaves (fresh)	73.77	70.31
*S. aqueum* Leaves (dried)	58.73	65.08
*P. foetida* Leaves (fresh)	78.13	75.38
*P. foetida* Leaves (dried)	66.67	67.74

### Correlation between phenolic content and antioxidant activity

**Figure 4:**
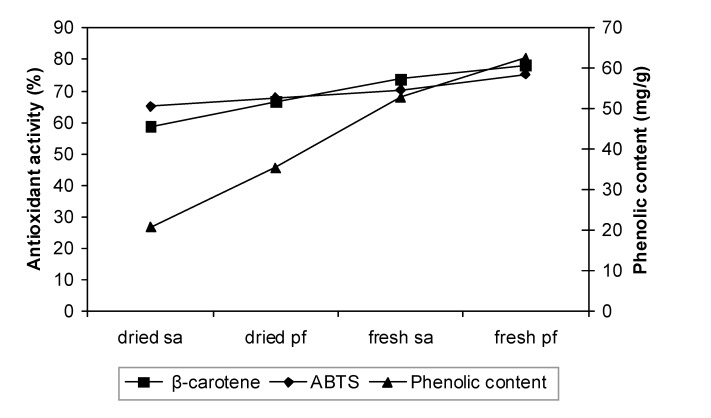
Correlation between phenolic content and antioxidant activity.

Some studies report a strong correlation between phenolic content and antioxidant activity in fruits, vegetables and grains [14] while other reports do not [12,16]. In this study the antioxidant activity of the plant extracts correlated well with the total phenolic content. A positive correlation was observed, whereby the antioxidant activity increased when the total phenolic content increased (Figure 4).

## Conclusions

This study clearly indicated that *P. foetida* and *S*. *aqueum*, both have high antioxidant activity. Fresh samples had higher phenolic contents and better antioxidant activity than did dried samples. There is not much difference in the total phenolic content between the leaves and twig of *P. foetida*. A good correlation between the β-carotene oxidation and ABTS methods was observed, with an R^2^ = 0.9878. The present study confirms that *P. foetida* and *S*. *aqueum* could be significant sources of natural antioxidant compounds that may have potent beneficial health effects.

## Experimental

### Preparation of samples

Plant materials were collected from trees growing in home gardens in Gelugor, Penang, Malaysia. Upon arrival at the laboratory, samples were washed with water to remove debris. The leaves and twigs of *P. foetida* and leaves of* S. aqueum* were stripped from the plants. Plant materials (500 g) were divided into fresh and dried samples. The dried samples were air dried at room temperature (30 °C) for 14 days until a constant weight was achieved. Fresh samples were air dried at room temperature (30 °C) for 24 hr and were immediately immersed in the solvents used for extraction.

### Preparation of extracts

All fresh and dried samples were extracted with methanol: water (1:10). The aqueous methanol solution was then filtered through Whatman No. 4 filter paper and the solvent was removed *in vacuo*. The crude extract was transferred into a 100 mL volumetric flask and ethanol was added up to the mark to prepare solutions at different concentrations (0.005 - 0.1 mg mL^-1^). The extracts were stored at -20 ºC. These crude leave extracts of *P. foetida* and of * S. aqueum* were subjected to ABTS free radical scavenging activity and antioxidant activity towards β-carotene oxidation, while all crude leaves and twig extracts were used for the analysis of total phenolic content.

### Antioxidant activity

### Coupled oxidation of β-carotene and linoleic acid

The β-carotene bleaching assays were conducted as previously described with slight modifications [21,22]. A mixture of β-carotene (60 mg, Sigma Chemical Co.), linoleic acid (1.0 g, Sigma Chemical Co.) and Tween^®^ 40 (20 mL, Sigma Chemical Co.) were dissolved in chloroform (20 mL, Merck). Chloroform was removed at 40 °C with a rotary evaporator. After evaporation, the mixture was immediately added to oxygenated distilled water (25 mL) to form an emulsion. The emulsion (25 mL) was transferred to test tubes containing extracts (1.0 mL) and the mixture was then gently mixed. One mL of the mixture was pipetted and mixed with 95% ethanol (5 mL) at 0 °C. Absorbance of the samples at 450 nm were measured in triplicates every 20 min. for a duration of 160 min. with a Hitachi U-2000 Spectrophotometer. The above procedure was repeated using DL-α-tocopherol (Sigma Chemical Co.) and quercetin (Sigma Chemical Co.) as standards. A blank solution without β-carotene was prepared containing the same concentration of sample. The total antioxidant activity was calculated based on the following equation:

AA = [1 – (*A_s_^0^* - *A_s_^160^*)/(*A_c_^160^* - *A_c_^160^*)] X 100

where *A*_s_^0^ is the absorbance of sample at 0 min, *A*_s_^160^ is the absorbance of sample at 160 min, *A*_c_^0^ is the absorbance of control sample at 0 min, and *A*_c_^160^ is the absorbance of control sample at 160 min.

### ABTS free radical scavenging activity

Radical scavenging activity was measured as previously described [23,24] with minor modifications. 2,2’-Azinobis(3-ethylbenzothiazoline-6-sulfonic acid (ABTS) was used as the free radical source and prepared by reacting 3.75 mM ABTS diammonium salt (Fluka) and 1.225 mM potassium persulphate (BDH chemicals) overnight at 30 °C. The mixture was diluted 10-fold with 99.5% ethanol (Merck) before use. The diluted ABTS radical solution (3.0 mL) was added to (*l*)-(+) ascorbic acid standards (0.005 g mL^-1^ – 0.1 mg mL^-^1, 1.0 mL, Merck), and the mixtures were incubated for 60 minutes. The absorbance at 414 nm was then measured at 30 ºC. The procedure was repeated with quercetin (Sigma Chemical Co.) standard, followed by fresh and dried *P. foetida* and *S. aqueum* extracts. A control sample (without antioxidants or extract), containing the same amount of ethanol and ABTS radical was prepared and measured daily. The scavenging ability of antioxidants was calculated according to the following equation :

ABTS scavenging activity (%) = [(A_0_ – A) / A_0_] x 100

where A_0_ is the absorbance of the control reaction and A is the absorbance in the presence of samples at 60 min.

### Quantitative determination of total phenolic content

The total phenolic content of the crude methanol extract was determined by using a modified Folin-Ciocalteu method with ferulic acid (Sigma Chemical Co.) as a standard [25]. Folin-Ciocalteu reagent (0.25 mL, Fluka) was added to methanolic extract solution of (1.0 mg mL^-1^, 10 mL), then 20% aqueous sodium carbonate solution (1.2 mL) was added and the tube vortexed and then incubated for 40 minutes. A blue color appeared and the absorbance was measured at 725 nm with a Hitachi U-2000 Spectrophotometer. All measurements were made in triplicates and the results expressed as mg of ferulic acid per gram of sample. 
